# An 8-model ensemble of CMIP6-derived ocean surface wave climate

**DOI:** 10.1038/s41597-024-02932-x

**Published:** 2024-01-20

**Authors:** Alberto Meucci, Ian R. Young, Claire Trenham, Mark Hemer

**Affiliations:** 1 Department of Infrastructure Engineering, The University of Melbourne, Parkville, Victoria 3010 Australia; 2 Climate Science Centre, CSIRO Environment, Aspendale, VIC 3195 Australia; 3 Climate Science Centre, CSIRO Environment, Black Mountain, ACT 2600 Australia; 4 Climate Science Centre, CSIRO Environment, Hobart, TAS 7001 Australia

**Keywords:** Projection and prediction, Physical oceanography

## Abstract

We present a global wind wave climate model ensemble composed of eight spectral wave model simulations forced by 3-hourly surface wind speed and daily sea ice concentration from eight different CMIP6 GCMs. The spectral wave model uses ST6 physics parametrizations and a global three-grid structure for efficient Arctic and Antarctic wave modeling. The ensemble performance is evaluated against a reference global multi-mission satellite altimeter database and the recent ECMWF IFS Cy46r1 ERA5 wave hindcast, ERA5H. For each ensemble member three 30-year slices, one historical, and two future emission scenarios (SSP1-2.6 and SSP5-8.5) are available, and cover two distinct periods: 1985–2014 and 2071–2100. Two models extend to 140 years (1961–2100) of continuous wind wave climate simulations. The present ensemble outperforms a previous CMIP5-forced wind wave climate ensemble, showing improved performance across all ocean regions. This dataset is a valuable resource for future wind wave climate research and can find practical applications in offshore and coastal engineering projects, providing crucial insights into the uncertainties connected to wind wave climate future projections.

## Background & Summary

Wind waves are ocean surface waves generated as a result of the wind blowing over the ocean surface. Wind waves, particularly their extreme estimates, play a crucial role in the accurate and efficient design of maritime structures^1^ and as a primary element of coastal erosion and flood risk assessment studies^2^. As the Earth is warming, the ocean surface wind climate is changing, and thus, the waves^3^. This is a major concern for growing coastal communities which are already facing challenges posed by sea level rise^4,5^. As a result, there has been growing interest in understanding how the wind wave climate is changing^6^ and how it will change in the future^7,8^.

One of the most common approaches to estimate wind wave climate projections is by using global spectral wave models^9^ forced by the Climate Model Intercomparison Project (CMIP) General Circulation Models (GCM) 10-meter surface wind speeds^10–12^. However, these projections are plagued by uncertainties^13^. First, our limited understanding of the nature of climate and the coarse temporal and spatial resolution of GCMs impact the performance of ocean surface wind speed projections^14,15^. The downscaling of these products to match the spectral wave model resolution adds further uncertainties around the wind forcing climate performance. In addition to this, even though spectral wave models perform well for global wind wave average statistics^16,17^, challenges persist in the representation of extreme wind waves, due to the limited spatial resolution of global wave models^14^ and the complex nature of atmosphere-ocean interactions at the ocean surface^18^. Another aspect that influences the range and amplitude of future climate predictions is internal climate variability. These are the natural fluctuations in the Earth’s climate system that occur independently of external influences such as changes in solar radiation or human-induced factors. In addition, future climate projections rely on greenhouse gas emission scenarios, and thus, depend on accurate and sophisticated predictions of future global society development. All these factors result in inter-model and intra-model uncertainties that, in a cascading effect, can accumulate and grow, making it increasingly difficult to predict future wind wave climate changes. This emphasizes the need for comprehensive research studies that leverage the developments in the representation of ocean surface wind speed within climate models, alongside the improvements of global spectral wave models, to enhance the representation of the past climate. The wide range of uncertainties also underscores the need for an ensemble approach when projecting future ocean wave climate. Ultimately, the utilization of more sophisticated and refined future emission scenarios may support a clearer understanding of the uncertainties associated with future projections.

In the past few years major developments have been achieved with the latest CMIP phase 6 (CMIP6)^19^ GCMs that demonstrate a better overall performance over previous CMIP generation GCMs as a consequence of higher temporal and spatial resolution, as well as improved physics^20,21^. In parallel with this, there have been significant developments in the spectral wave model representation of wind waves. The main advances have been introduced by two so-called Source Terms (ST) wave physics parametrizations commonly referred to as ST4^22^ and ST6^23^. Through the analysis of global remote sensing measurements^24,25^ and laboratory and field measurement campaigns^26,27^, ST4 and ST6 have brought forth advancements in various aspects of wind wave modeling, ranging from atmosphere-ocean interactions to wave propagation and wave dissipation. Using such updated products and wave models, we expect a better overall performance of past climate representation, as this paper will later confirm. Yet, future projections add another layer of uncertainty. In this space, the future emissions scenarios have been redefined into the Shared Socio-economic Pathways (SSPs) to include aspects such as adaptation and mitigation strategies^28^. This means that updated emissions scenarios can be used to evaluate the ocean surface wind speed projections and ultimately the waves. Whether this brings a significant improvement in reducing the uncertainties is still to be assessed.

The objective of the present wind wave climate ensemble is to leverage all these advances in the field of wind-wave climate modeling, and improve on spatial and temporal resolution, to provide a better overall ensemble performance in comparison to similar previous generation CMIP5-derived wave climate models^29^. The performance of the CMIP6 and CMIP5 datasets is assessed by comparing them to reference observational^30^ and hindcast data^31^ in order to understand the improvements in representing historical wave climate patterns. The objective of this paper is to support an informed use of the dataset for future ocean wind wave climate studies.

## Methods

The present global wave climate model ensemble is produced with WAVEWATCH III v6.07.1^32^ (hereafter WW3), a state-of-the-art spectral wave model that solves the action density balance equation as a function of wavenumber and direction. WW3 is forced by 3-hourly eastward (uas) and northward (vas) components of the 10-meter surface wind speed and the daily sea ice concentration (siconc) from eight CMIP6 GCMs (Table 1): ACCESS-CM2^33^ (ACM2), AWI-CM-1-1-MR^34^ (AWI), CMCC-CM2-SR5^35^ (CMCC), EC-Earth3^36^ (EC3), IPSL-CM6A-LR^37^ (IPSL), KIOST-ESM^38^ (KIOST), MPI-ESM1-2-LR^39^ (MPI), MRI-ESM2-0^40^ (MRI). The GCMs datasets are publicly available at the Earth System Grid Federation (ESGF) node (https://esgf-node.llnl.gov/search/cmip6/). In this project we used replicas of the GCMs variables from the Australian National Computational Infrastructure (NCI). Throughout the paper we refer to the abbreviations in brackets when referring to the specific GCMs. We add WW3 in front of the GCM abbreviation if we refer to the wave model run forming part of the present wave climate model ensemble (e.g., WW3/ACM2).

**Table 1:** CMIP6 GCMs r1i1p1f1 3-hourly eastward (uas) and northward (vas) components of the 10-meter surface wind speed and daily sea ice concentration (siconc) forcing chosen to run the wave climate ensemble.

Model	Variable ID	Grid	Global Grid Resolution
ACCESS-CM2^33^ (ACM2)	uas, vas	MetUM-HadGEM3-GA7.1 N96	192 × 144
	siconc	GFDL-MOM5 tripolar	360 × 300
AWI-CM-1-1-MR^34^ (AWI)	uas, vas	ECHAM6.3.04p1 T127/L95 gaussian	384 × 192
	siconc	FESOM 1.4 unstructured	830305 wet nodes
CMCC-CM2-SR5^35^ (CMCC)	uas, vas	CAM5.3 Finite Volume regular grid	288 × 192
	siconc	NEMO3.6 ORCA1 tripolar	362 × 292
EC-Earth3^36^ (EC3)	uas, vas	IFS cy36r4 TL255/L91 linearly reduced gaussian	512 × 256
	siconc	NEMO3.6 ORCA1 tripolar	362 × 292
IPSL-CM6A-LR^37^ (IPSL)	uas, vas	LMDz regular C-grid	144 × 143
	siconc	Native ocean tripolar	105000 ocean cells
KIOST-ESM^38^ (KIOST)	uas, vas	GFDL-AM2.0 cubed sphere	192 × 96
	siconc	GFDL-MOM5.0 tripolar	360 × 200
MPI-ESM1-2-LR^39^ (MPI)	uas, vas	ECHAM6.3 spectral T63	192 × 96
	siconc	MPIOM1.63 bipolar	256 × 220
MRI-ESM2-0^40^ (MRI)	uas, vas	MRI-AGCM3.5 TL159/L80	320 × 160
	siconc	MRI.COM4.4 tripolar	360 × 364

**Fig. 1:**
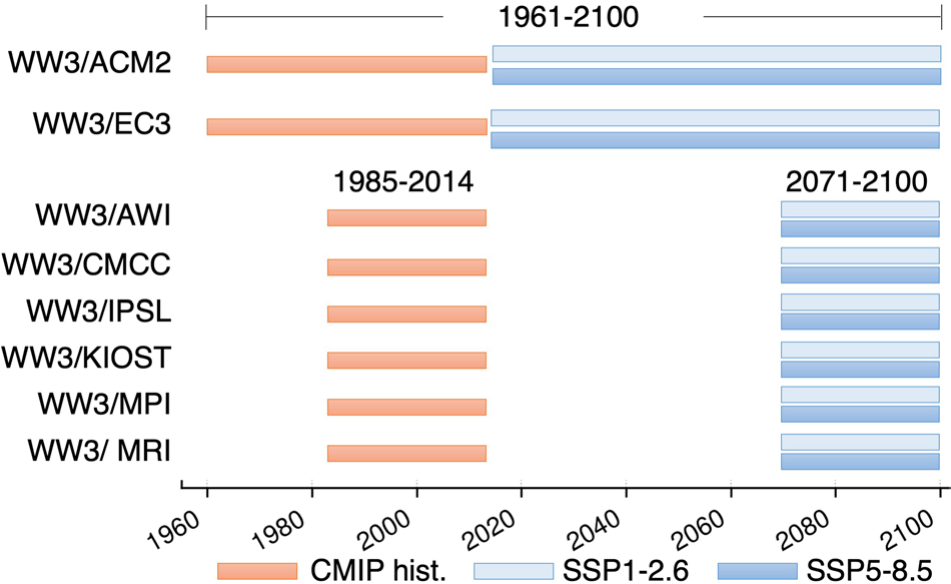
Gantt chart (time duration) of the WW3 8-model wave climate ensemble. In orange, the CMIP historical runs, in light blue the SSP1-2.6 mid-emission scenario, and in blue the SSP5-8.5 high-emission scenario. Note that, the WW3/ACM2 and the WW3/EC3 models were also run for 140 years of continuous wave climate projections^11^. The rest of the models composing the ensemble are run for the CMIP historical 1985–2014 and the SSP1-2.6 and SSP5-8.5 2071–2100 30-year time slices.

Figure 1 shows the time duration of the WW3 climate runs. For each GCM we selected two 30-year time slices (1985–2014 and 2071–2100). Two WW3 runs, WW3/ACM2 and WW3/EC3, were also performed across a continuous 140-year period from 1961 to 2100^11^. For each GCM we run the CMIP historical experiment (1985–2014), and two Shared Socio-Economic Pathways (SSP), the mid-emission scenario, SSP1-2.6, and the high-emission scenario, SSP5-8.5^28^ for the period from 2071 to 2100. Despite being a very unlikely future^41^, we use the SSP5-8.5 scenario to facilitate the comparison with previous generation CMIP wave climate ensembles. Also, as the climate system feedbacks are still not well understood, we deem the SSP5-8.5 run still valuable as a “worst case” scenario to communicate to the users an idea of what is the upper end of the future possible scenarios spectrum.

The selection of GCMs conformed to the following criteria:

- The GCMs availability of 3-hourly wind speed and daily sea ice concentrations. The objective here is to have a temporal resolution high enough to capture the variability of ocean storms. This ensures a better depiction of extremes in the resulting modeled wind wave time series.
- The performance of the GCMs’ 10-meter surface wind speed against the reference European Re-Analysis v5 (ERA5)^42^ wind speed^11^.
- The alignment with the Australian Climate Service (ACS) CMIP6 GCMs selection for the World Climate Research Programme (WCRP) Coordinated Regional Climate Downscaling Experiment (CORDEX) in the Australian region^43^.

We selected the r1i1p1f1 (r: realization, i: initialization, p: physics, f: forcing) variant for all GCMs. We ran a preliminary test of the impact of choosing different ensemble realizations (r1, r4, and r5) on the surface wind speed climatology of the ACM2 GCM (Figure S1). We found only minor differences in the global average mean (Figures S1a–c, S2a–c) and 90^th^ percentile (Figures S1d–f, S2d–f) wind speed climatology that have no major impact on the wind wave climate statistics. However, this test is not exhaustive, and further research should be dedicated to evaluating not only different realizations (r) but also different initialization (i), physics (p), and forcing (f) of each GCM^44^. Future studies could also assess the impacts of internal climate variability on extremes trends computed from different GCMs realizations.

A three-grid system^45^ was implemented in WW3 to model the polar regions^11,17^. A regular grid spans from 55°N to 55°S with a nominal resolution of 55 km and the two curvilinear grids for the polar regions with a nominal resolution of 34 km at 70°N (70°S)^45^. The WW3 runs used the default calibration of the ST6 source term physics package^46^, also known as observation-based physics. The wave physics parametrizations in ST6 were derived from extensive field measurement campaigns^26,47,48^. A possible alternative would be to use the ST4 source term parametrization scheme^22^. The ST4 and ST6 parametrizations have been demonstrated to be the best performing physics packages available in terms of global average wind wave climate statistics^49,50^. Both ST4 and ST6 are currently implemented in major international oceanic and atmospheric centers^31,51–53^. The selection of the ST6 scheme is in line with the newly developed Australian operational wave forecast model^52^.

A fundamental aspect of global wind wave modeling is the calibration of the wave model to the wind forcing field^54^. This is done in ST6 through a bulk adjustment of the drag coefficient (CDFAC) following the Hwang *et al*.^55^ wind stress formulation. This formula acts as a cap to the wind input transferred to the waves. A higher CDFAC value means that a less restrictive limit is applied to the wind input, and, as such, the model may transfer more energy to the waves. The impact of two CDFAC bulk adjustments (CDFAC = 1.0 and CDFAC = 1.08) is shown in Meucci *et al*.^11^. CDFAC = 1.0 is the default calibration^46^ to the Climate Forecast System Reanalysis (CFSR)^56^ surface wind speed field. CDFAC = 1.08 covers the range of test runs performed for an ST6 global wave model forced by ERA5 surface wind speed^17^. The Liu *et al*.^17^ hindcast has a similar three-grid structure and physics parametrizations to the present wave climate ensemble. Note that the CDFAC is a crude adjustment and, as such, only changes the wave climatology to higher or lower values across the whole distribution of wind wave values, i.e., from the minimum, to the average, to the extreme values. As the aim of this project was to produce a wave climate model ensemble, running a specific calibration to each GCM’s wind field would have only introduced an additional source of uncertainty for future comparisons. As such, for consistency, and to facilitate the comparison of each wave climate model composing the ensemble, we set the same wind speed bulk adjustment calibration (CDFAC = 1.00) for all model runs.

## Data Records

The present CMIP6-derived wave climate ensemble has been published in two phases: Phase 1^57^ and Phase 2^58^. The combined dataset^59^ can be accessed through the data access portal (http://hdl.handle.net/102.100.100/601698). The webpage will redirect the user to the THREDDS server page to download the dataset.

The output of each model is re-gridded globally over a regular grid (glout) with 0.5° spatial resolution (from 89°N to 90°S). The model masks the values around the North Pole to avoid geometrical singularities^11^. The outputs are divided into field and point outputs. The field outputs, also referred to as integral parameters, are shown in Table 2. The point outputs consist of directional spectra with resolution of 50 frequencies and 36 directions extracted at specific locations around the globe^11^. The locations are, globally on a 10° grid, and locally for a ring around the Australian continent, and a box around the South-East Australian domain (Table 2).

**Table 2:** Wind wave climate ensemble WW3 model outputs.

	Variable	ID
Integral parameters:	10-meter wind speed Eastward and Northward components	wnd
	Significant Wave Height, *H*_s_	hs
	Significant wave height partitions: Wind-sea, *pH*_s, 0_, Swell 1, *pH*_s, 1_, Swell 2, *pH*_s, 2_	phs0, phs1, phs2
	Mean wave direction, *θ*	dir
	Peak wave direction, *θ*_*p*_	dp
	Spectral second moment mean wave period, *T*_m, 02_	t02
	Peak frequency, *f*_*p*_ = 1/*T*_*p*_	fp
	Wave energy flux, *C*_*g*_*E*	cge
Directional spectra:	10° × 10° global grid	g10d
	Australian continent boundary	AUSring
	South-East Australia boundary	VCboun

### Directories

The dataset is organised in three directories: historical, ssp126, and ssp585. Each of these directories has subdirectories with each of the model runs. The WW3/ACM2 and the WW3/EC3 have two different wind calibration results available corresponding to a CDFAC of 1.0 and 1.08^11^. The ww3_ounf_glout subdirectory contains the NetCDF files described below.

### Filenames

All data are available as NetCDF files. The folder ww3_ounf_glout contains the integral parameter NetCDF (.nc) outputs divided by months and parameter following the nomenclature:

ww3.YYYYMM_var.nc

where YYYY is the year, MM is the month, and var is the name of the integral parameter variable as shown in Table 2. The directional spectra point outputs are stored in a similar structure and available upon request. These are divided in the three groups indicated in Table 2 and the files follow this nomenclature:


ww3.YYYYMM_spec.nc


each file contains the spectral information at all locations.

## Technical Validation

In this section, we evaluate the present wave climate ensemble performance to facilitate informed usage of the dataset for future wave climate studies. We evaluate the ensemble average surface wind speed, ${\overline{U}}_{10}$, and significant wave height, ${\overline{H}}_{{\rm{s}}}$, climatology performance in comparison with multi-mission satellite altimeter observations binned over a 2° × 2° global regular grid^30^ over a 23-year period, from 1992 to 2014. This is an optimal overlapping period as it excludes the sparse satellite observations available before 1992.

We then extend the ensemble validation to other integral parameters, namely the mean wave period, ${\overline{T}}_{{\rm{m,02}}}$, the mean wave direction, $\overline{\theta }$, and the wave energy flux, $\overline{{C}_{g}E}$. To do so, we compare the present ensemble with the European Centre for Medium-Range Weather Forecasts (ECMWF) Integrated Forecasting System (IFS) Cy46r1 ERA5 wave Hindcast, ERA5H^31^. This hindcast is preferred over the reanalysis ERA5, as this wave model runs the updated ST4 physics parametrizations^31^, and it does not assimilate wave height observations, removing any possible non-homogeneities^60,61^. These aspects make ERA5H a more suitable product to perform a direct comparison to the present wind wave climate model ensemble. In contrast to the satellite validations, all the comparisons with the ERA5H run are conducted over the full 30-year ensemble historical time slices, from 1985 to 2014.

In addition, we conducted a comparison between the current CMIP6 wave climate ensemble and a similar previous CMIP5 generation ensemble^29^. In this case, the analysis involves two distinct time periods, the 1985–2014 for the present CMIP6 ensemble and the 1979–2004 for the previous CMIP generation ensemble. The objective is to assess if there has been any improvement over a similar previous generation wave climate model ensemble. Note that, we considered only seven of the eight climate models composing the CMIP5 ensemble, removing CNRM-CM5 model run due to its poor performance^29^. The datasets, the time windows, and the integral parameters evaluated in each comparison are summarized in Table 3.

**Table 3:** Reference datasets, time slices, and parameters used to evaluate the present CMIP6-derived wind wave climate ensemble performance.

Reference Dataset	Period	CMIP6 period used for comparison with ref. dataset	Parameters
Multi-mission satellite altimeters	1992–2014	1992–2014	*H*_s_, *U*_10_
ERA5 wave hindcast, ERA5H	1985–2014	1985–2014	${H}_{{\rm{s}}}^{}$, *T*_m, 02_, *θ*, *C*_*g*_*E*
CMIP5-ensemble	1979–2004	1985–2014	*H*_s_, *T*_m, 01_ (T_m, 02_ for CMIP6), *θ*

Note that, the *T*_m, 01_ parameter is used to evaluate the CMIP5 ensemble M-score performance. The comparison of the CMIP6 and the CMIP5 ensemble is done comparing different time windows against ERA5H, 1985–2014 for CMIP6 and 1979–2004 for CMIP5.

### Wind wave climate global spatial distribution

**Fig. 2:**
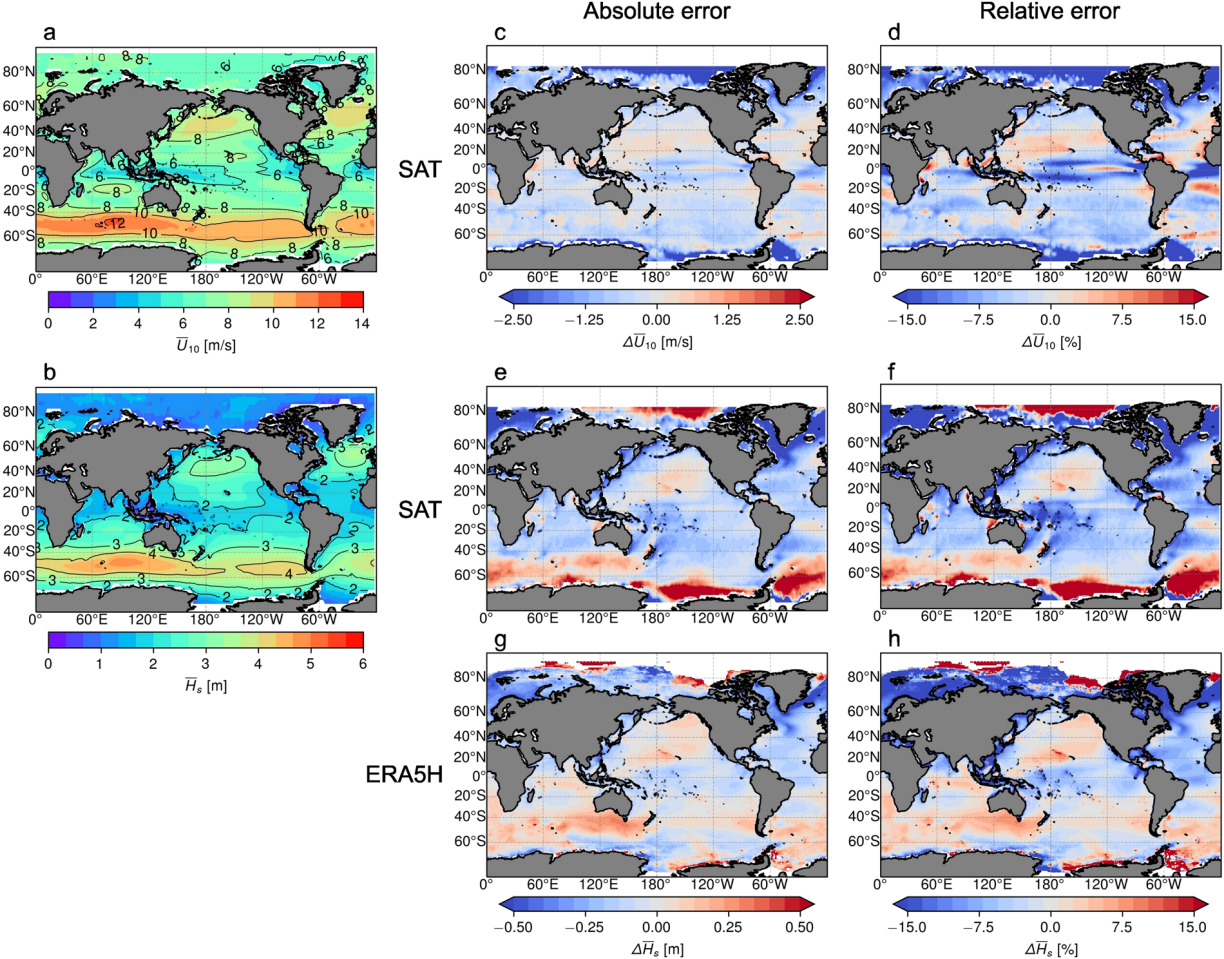
Multi-Model Ensemble (MME) spatial distribution performance against satellite altimeter (SAT) and ERA5H hindcast. The 1985–2014 (**a**) *U*_10_ and (**b**) *H*_s_ MME average climatology. (**c,d**) The MME 1992–2014 *U*_10_ absolute and relative error in comparison with the SAT climatology. (**e,f**) Same as (**c,d**) but for *H*_s_. (**g,h**) The MME 1985–2014 *H*_s_ absolute and relative error in comparison to ERA5H.

Figure 2 shows the comparison of the Multi-Model Ensemble (MME) average 10-meter surface wind speed, ${\overline{U}}_{10}$, and significant wave height, ${\overline{H}}_{{\rm{s}}}$, climatologies with the satellite altimeter measurements (SAT) at 2° × 2° resolution, and the ERA5H at 0.5° × 0.5° resolution. The MME statistics are computed using a ‘model democracy’ approach where each model is equally weighted. Figure 2a,b show the MME ${\overline{U}}_{10}$ and ${\overline{H}}_{{\rm{s}}}$ averages over the 1985–2014 period. Figure 2c,e,g show the absolute error, whereas Fig. 2d,f,h show the relative error percentages. The two comparisons (altimeter and ERA5H model) show different aspects of the wave climate ensemble performance. The high model bias in the polar regions seen in the comparison with the SAT dataset is due to the sparse satellite measurements available in the region. If we exclude the polar regions, the highest differences between the MME and SAT ${\overline{U}}_{10}$ climatologies are found in the Tropical and Equatorial regions. In contrast to this, the highest biases in the modeled ${\overline{H}}_{{\rm{s}}}$ are found at higher latitudes. The different spatial distribution of the biases between the wind speed and the wave height modeled demonstrates the different nature of these variables. Also, Fig. 2e,f show clear negative model ${\overline{H}}_{{\rm{s}}}$ bias in the lee of the Pacific Islands and the Southern Hemisphere (SH) continents. The same behaviour is not seen in the comparison with the ERA5H model. These differences are most likely a result of a well known wave model problem in representing directional spreading and wave diffraction, which results in less energy being propagated around the islands and the SH continents in both our models and the ERA5H reference model.

### Single model performance relative to the ensemble

**Fig. 3:**
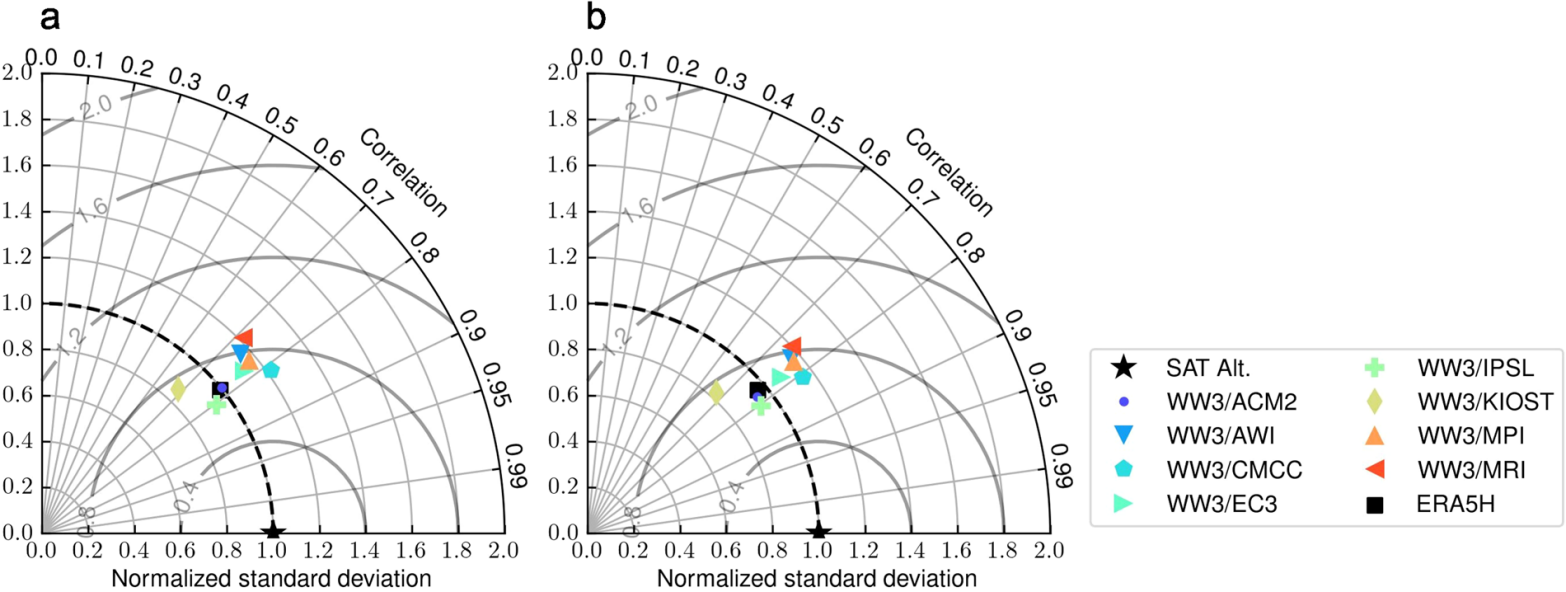
The 1992–2014 (**a**) *H*_s_ and (b) ${H}_{{\rm{s}}}^{p90}$, mean and 90th percentile significant wave height climatology in relation to the 2° × 2° satellite altimeter (SAT) (represented by a black star) for each WW3/GCM model ensemble member. The ERA5H performance in relation to the SAT dataset over the same period is marked with a black square. The x and y axis represent the standard deviation normalized by the reference SAT standard deviation (*σ*_*SAT*_). The SAT statistic is placed at the x-axis unit as a reference point. The radial position on the graph represents the Pearson correlation coefficient calculated for each instance of WW3/GCM and ERA5H models against the SAT dataset. The concentric grey circles centered in the black star (x-axis unit) illustrate the Root Mean Square Error (RMSE) values in comparison to SAT.

Following the IPCC AR4 climate model evaluation framework^62^ and using the Program for Climate Model Diagnosis and Intercomparison (PCMDI) Metrics Package (PMP)^63^, we assess the performance of each wave climate model relative to the overall MME statistics. Figure 3 shows the Taylor plot^64^ of the average ${\overline{H}}_{{\rm{s}}}$ (Fig. 3a), and the 90th percentile ${H}_{{\rm{s}}}^{p90}$ (Fig. 3b), against the reference multi-mission satellite altimeter statistics for the period 1992–2014. The plots also show the performance of the ERA5H hindcast (black square). The Taylor diagram consists of the standard deviation of each model normalised by the reference standard deviation, that is, the standard deviation of the satellite altimeter measurements. The Pearson correlation is shown as the radial coordinate, and the concentric circles centered in the black star show the RMSE values compared to the reference satellite altimeter dataset. For the comparison, the climatological monthly means for each ensemble member and ERA5H are re-gridded to the 2° × 2° multi-mission satellite altimeter climatological monthly statistics resolution. This is, twelve values for each grid cell over the 1992–2014 period, excluding the year 1991 due to limited satellite observations. To remove any spurious values due to the sparse satellite altimeter measurements in the polar regions, the comparison is performed over a domain masked from 60°N to 60°S.

Figure 3a shows that the wave climate models and ERA5H correlate similarly to the SAT dataset. Similar correlation values are found for the climatological monthly 90th percentile statistics, ${H}_{{\rm{s}}}^{p90}$ (Fig. 3b). The standard deviations of the WW3/ACM2 and WW3/IPSL climate models best compare with the reference SAT standard deviation. If the same analysis is performed over the 1992–2014 monthly mean time series (23 years × 12 values for each grid cell, Figure S3a) then, as expected, the wave climate models show lower correlation with the SAT dataset in comparison with ERA5H. This is because wave climate models are not in sync with historical weather oscillations. Correlation values further reduce for the 90th percentiles statistics, ${H}_{{\rm{s}}}^{p90}$ (Figure S3b).

**Fig. 4:**
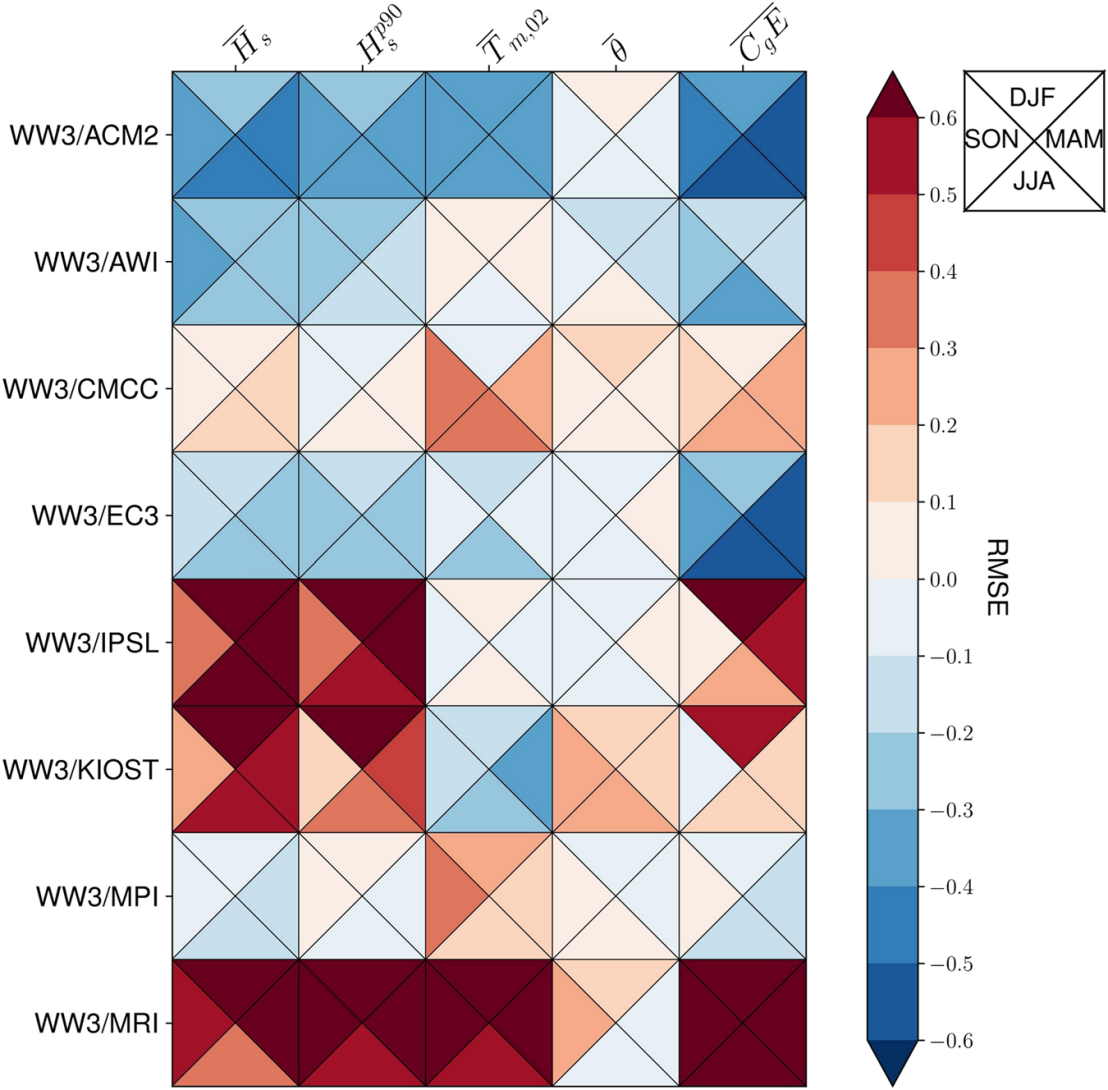
The 1985–2014 RMSE portrait diagram^63^ of relative seasonal (DJF, MAM, JJA, and SON) error metrics for each WW3/GCM ensemble member in relation to ERA5H. Each WW3/GCM performance is plotted relative to the median of the ensemble (*S*_*i*_ in Eq. 1). Each row is a WW3/GCM ensemble member, and each column a different integral parameter output, namely: the average and the 90th percentile significant wave height, *H*_s_ and ${H}_{{\rm{s}}}^{p90}$, the mean second-order spectral wave period, *T*_m, 02_, the mean wave direction, *θ*, and the wave energy flux, *C*_*g*_*E*. The models with a smaller (larger) RMSE than the ensemble median RMSE are shown in blue (red). Overall, the WW3/ACM2 is the best performing model and the WW3/MRI is the ensemble member with the poorest performance.

Figure 4 shows the PCDMI PMP portrait plot^63^ computed for each of the eight models composing the wind wave climate ensemble and for five integral parameter outputs. These are: the mean significant wave height, ${\overline{H}}_{{\rm{s}}}$, the 90th percentile significant wave height, ${H}_{{\rm{s}}}^{p90}$, the mean second order spectral mean wave period, ${\overline{T}}_{{\rm{m,02}}}$, the mean wave direction, $\overline{\theta }$, and the wave energy flux, $\overline{{C}_{g}E}$ (Table 3). The wave direction statistics are calculated using a unit vector mean approach. This involves decomposing the wave direction (output labelled as “dir” from WW3) into its eastward and northward vector components. The average of each component is determined. Subsequently, the average eastward and northward components are translated back into an overall mean direction. This entire process is carried out for each grid cell. The RMSE statistics are then computed spatially based on the mean direction values obtained for each grid cell. The portrait plot is computed from the RMSE statistics of each climatological monthly mean variable (12 values per each grid cell) relative to the ERA5H dataset. In each column the model *RMSE*_*i*_ is normalised by the ensemble median RMSE to plot the *S*_*i*_ statistic as shown in Eq. 1.

1 $${S}_{i}=\frac{RMS{E}_{i}}{Median(RMS{E}_{i})}\quad {\rm{where}}:RMS{E}_{i}=\frac{1}{n\cdot m}\mathop{\sum }\limits_{j,k}^{n,m}RMS{E}_{j,k}\quad j,k:{\rm{latitude}},{\rm{longitude}}$$

As such, Fig. 4 shows the performance of each model relative to the overall ensemble performance for each integral parameter. The models that overperform (underperform) in respect to the overall ensemble are shown in blue (red). Each *S*_*i*_ statistic is computed by season and shown as DJF, MAM, JJA, and SON triangles. In this way, Fig. 4 also shows each model performance in reproducing the wind wave climate seasonality. Figure 4 shows that the WW3/IPSL, WW3/KIOST and the WW3/MRI have poorer performance statistics than the rest of the models. The best performing models of the ensemble are the WW3/ACM2 and the WW3/EC3 models. These are also the models that have been run continuously for 140 years (Fig. 1).

### Improvements over previous CMIP generations

We further analyse the performance of the MME average climatology by ocean regions. To do so, we employ the non-dimensional Arcsin-Mielke measure (M-score)^65^. This is, the RMSE non-dimensionalized by the spatial variance of the field. We use this metric as it allows a direct comparison of the present CMIP6 8-model ensemble with the previous generation Australian CMIP5 wave climate 7-model ensemble^29^ (the poorly performing CNRM-CM5 was excluded for a fair comparison). The M-score is calculated between the CMIP5 and the present CMIP6 ensemble models and the ERA5H model. The metric is computed after we interpolate the ERA5H results to the CMIP model resolution.

**Fig. 5:**
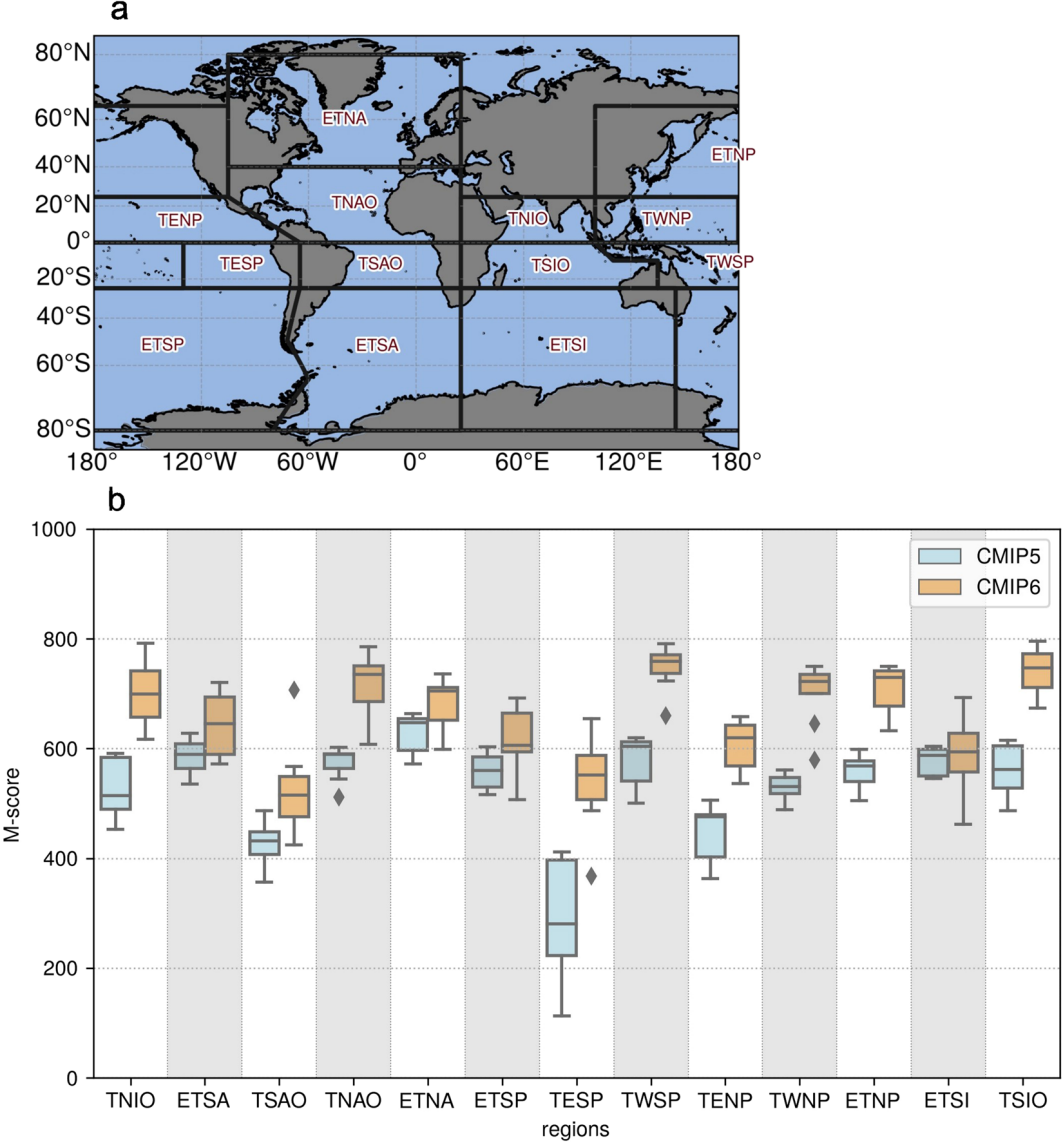
The present 1985–2014 CMIP6 and the 1979–2004 CMIP5^29^ wave climate ensembles performance in relation to ERA5H. (**a**) Alves^66^ reference ocean climatic regions: Tropical North Indian Ocean (TNIO), Extra-Tropical South Atlantic (ETSA), Tropical South Atlantic Ocean (TSAO), Tropical North Atlantic Ocean (TNAO), Extra-Tropical North Atlantic (ETNA), Extra-Tropical South Pacific (ETSP), Tropical Eastern South Pacific (TESP), Tropical Western South Pacific (TWSP), Tropical Eastern North Pacific (TENP), Tropical Western North Pacific (TWNP), Extra-Tropical North Pacific (ETNP), Extra-Tropical South Indian (ETSI), and Tropical South Indian Ocean (TSIO). (**b**) The M-score^65^ metrics by ocean climatic regions for the CMIP5 (light blue) and the CMIP6 (orange) ensemble members. Each WW3/GCM M-Score is computed by averaging the M-score of three integral parameter outputs: *H*_s_, *T*_m, 02_ (*T*_m, 01_ for CMIP5), and *θ*. The results are clustered into a box-plot that shows the median ensemble M-score, the Inter-Quartile Range and the minimum and maximum M-score values of each ensemble member.

The performance of the CMIP5 and CMIP6 MMEs in comparison with ERA5H is shown as M-score box-plots in Fig. 5 for the ocean climatic regions defined by Alves^66^. Each box-plot represents the distribution of the M-score results for each model composing the ensemble. The box plots shown are the average M-score values of three integral parameters: the significant wave height, *H*_s_, the mean period, *T*_m, 02_ (*T*_m, 01_ for the CMIP5 ensemble^29^), and the mean wave direction, *θ*. A value of 1000 means that the CMIP model is equal to the ERA5H reference dataset. Figure 5b shows that the present CMIP6 ensemble outperforms the previous generation CMIP5 ensemble in every ocean region depicted in Fig. 5a. This is due to multiple reasons, the most important being the updated GCMs wind forcing, and the updated wave model physics parametrizations and resolution. In Figure S4 of the Supplementary Material we show the performance of the present CMIP6 MME for the latest IPCC AR6 ocean climatic region classification^67^. The low M-score values in the Arctic Ocean are due to the high variability and poor performance of some GCMs in representing the sea ice concentration in the region^68^. In particular, we note a warm bias in the CMCC model and a cold bias in the KIOST model in the Arctic region. High intra-ensemble variability in the sea ice concentration is also found in the Antarctic Region^69^.

### Potential future applications

**Fig. 6:**
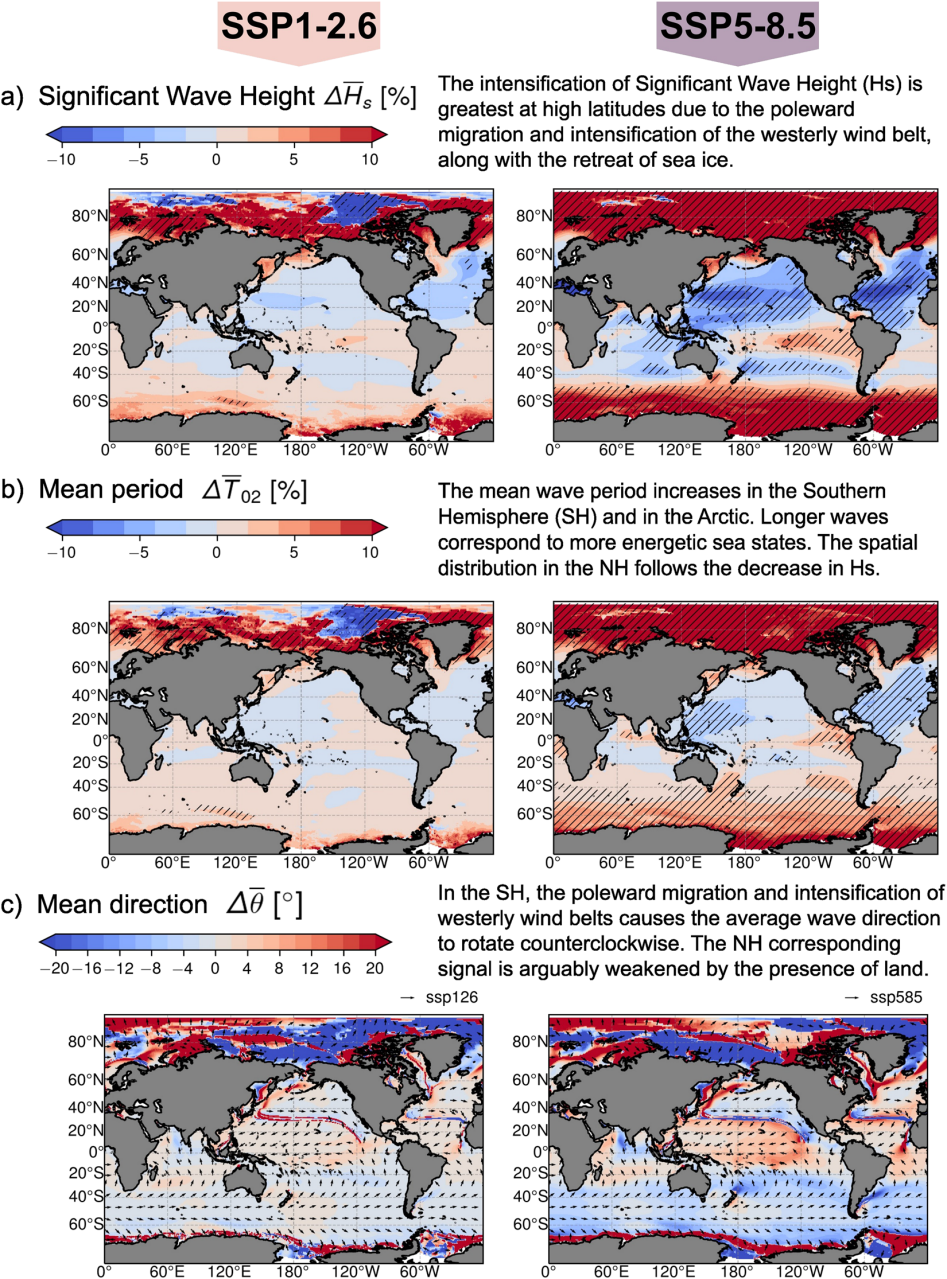
Potential applications of the present CMIP6 wave climate model ensemble. Global future projected wind-wave climate changes. The Δ in each panel refers to the difference between the end of the 21^st^ century (2071–2100) and the historical (1985–2014) climatology. The figure shows the SSP1-2.6 (left column) and the SSP5-8.5 (right column) future projected MME percentage changes of: (**a**) the significant wave height, $\Delta {\overline{H}}_{{\rm{s}}}$, (**b**) the mean second-order spectral wave period, $\Delta {\overline{T}}_{{\rm{m,02}}}$, and (**c**) the mean wave direction, $\Delta \overline{\theta }$. A ‘model democracy’ approach was followed to compute the MME average statistics where each model is equally weighted. The hatching in (**a,b**) defines where the $\Delta {\overline{H}}_{{\rm{s}}}$ and $\Delta {\overline{T}}_{{\rm{m,02}}}$ changes are statistically significant. The change is defined statistical significant if it surpasses the ensemble average SSP1-2.6 and SSP5-8.5 projections inter-annual variability (Figures S5b,c, S6b,c). The arrows in (**c**) show the 2071–2100 future projected average mean wave directions.

To demonstrate the potential applications of the present dataset, we show in Fig. 6 the MME future projected changes for a) ${\overline{H}}_{{\rm{s}}}$, b) ${\overline{T}}_{{\rm{m,02}}}$, and c) $\overline{\theta }$. Changes are computed for the SSP1-2.6 and the SSP5-8.5 scenarios comparing the average statistics for 30-year time slices: the 1985–2014 historical time slice and the end of the 21^st^ century 2071–2100. The Δ in Fig. 6 represent the difference between the MME average statistic of the end of the 21^st^ century and the historical time slice using a model democracy approach. In this context, each model is accorded equal weight, and no bias correction is implemented. Note that, these plots serve only as illustrations for potential applications of the present CMIP6 wind wave climate ensemble. Users should employ their preferred bias correction methodology or weighting approach when estimating the magnitude of changes from the ensemble.

In Fig. 6a,b, the positive values in red depict an increase in the average ${\overline{H}}_{{\rm{s}}}$ (${\overline{T}}_{{\rm{m,02}}}$) climatology. The present wind wave climate ensemble confirms the already observed increasing wind wave climate intensity at the high latitudes (Fig. 6a,b). This is due to the poleward movement and intensification of the westerly wind belts, together with the sea ice retreat in the polar regions. The poleward movement also causes a counterclockwise change in mean wave direction (blue) as shown in Fig. 6c.

The hatching in Fig. 6a,b defines where the changes are statistically significant for the present model democracy approach. In this case we define the change statistical significant if it surpasses the average inter-annual variability of the ensemble SSP1-2.6 and SSP5-8.5 projections respectively. The inter-annual variability is calculated as the average of the standard deviations of each ensemble member’s 30-year time series of yearly means. This information is presented in Figure S5a–c for ${\overline{H}}_{{\rm{s}}}$ and Figure S6a–c for ${\overline{T}}_{{\rm{m,02}}}$. To provide a tool for interpreting and understanding the statistical significance of the wind wave climate ensemble changes, we also computed the inter-member variability as the standard deviations of the ensemble member global climatological means. The corresponding results are detailed in Figure S5d–f for ${\overline{H}}_{{\rm{s}}}$ and Figure S6d–f for ${\overline{T}}_{{\rm{m,02}}}$.

Further research should be focusing on assessing the extent of uncertainties, particularly at the extremes. Additionally, there is a need to understand the potential implications of changes, both in mean values and extremes, at the regional scale. This evaluation is crucial for informing decisions related to coastal erosion, as well as the engineering design of coastal, offshore structures, and marine operations.

### Supplementary information

Supplementary Information

## Data Availability

The wind wave climate ensemble was produced using WAVEWATCH III v6.07.1^32^ release available at https://github.com/NOAA-EMC/WW3. The wind wave climate ensemble performance was evaluated through the combined use of the Climate Data Operator (CDO) suite^70^ and the PCMDI Metrics Package (PMP) Python library, publicly available at https://github.com/PCMDI/pcmdi_metrics.git.
