# Supplementary material for: An 8-model ensemble of CMIP6-derived ocean surface wave climate
Source: Sci Data. 2024 Jan 20;11:100. doi: 10.1038/s41597-024-02932-x (PMC10799940; doi:10.1038/s41597-024-02932-x)
Supplement: Supplementary file 1 — Supplementary Information [file 41597_2024_2932_MOESM1_ESM.pdf]

# Supplementary Information: An 8-model ensemble of CMIP6-derived ocean surface wave climate

**Alberto Meucci[1,2,*], Ian R. Young[1], Claire Trenham[3], and Mark Hemer[4]**

[1]Department of Infrastructure Engineering, The University of Melbourne, Parkville, Victoria 3010, Australia
[2]Climate Science Centre, CSIRO Environment, Aspendale, VIC 3195, Australia
[3]Climate Science Centre, CSIRO Environment, Black Mountain, ACT 2600, Australia
[4]Climate Science Centre, CSIRO Environment, Hobart, TAS 7001, Australia
[*]corresponding author: Alberto Meucci (alberto.meucci@unimelb.edu.au)

## ABSTRACT

We present a global wind wave climate model ensemble composed of eight spectral wave model simulations forced by 3-hourly surface wind speed and daily sea ice concentration from eight different CMIP6 GCMs. The spectral wave model uses ST6 physics parametrizations and a global three-grid structure for efficient Arctic and Antarctic wave modeling. The ensemble performance is evaluated against a reference global multi-mission satellite altimeter database and the recent ECMWF IFS Cy46r1 ERA5 wave hindcast, ERA5H. For each ensemble member three 30-year slices, one historical, and two future emission scenarios (SSP1-2.6 and SSP5-8.5) are available, and cover two distinct periods: 1985–2014 and 2071–2100. Two models extend to 140 years (1961–2100) of continuous wind wave climate simulations. The present ensemble outperforms a previous CMIP5-forced wind wave climate ensemble, showing improved performance across all ocean regions. This dataset is a valuable resource for future wind wave climate research and can find practical applications in offshore and coastal engineering projects, providing crucial insights into the uncertainties connected to wind wave climate future projections.

## List of Figures


S1 Evaluation of the 10-meter surface wind speed, $U_{10}$, climatology from different realizations of the ACCESS-CM2 GCM 1985–2014 CMIP historical experiment. (a,b,c) The average wind speed climatology difference, $\Delta\overline{U}_{10}$. (d,e,f) The 90$^{\text{th}}$ percentile wind speed climatology difference, $\Delta U_{10}^{p90}$. (a,d) $\Delta$ = r4i1p1f1 - r1i1p1f1, (b,e) $\Delta$ = r5i1p1f1 - r1i1p1f1, (c,f) $\Delta$ = r5i1p1f1 - r4i1p1f1. The hatching in (a, b, c) indicates statistically significant differences, where the p-value is less than 0.05, as determined by a Student t-test. . . . . . . . . . . . . . . . . . . 3

S2 Same as Figure S1 but for percentage changes. (a,b,c) The average wind speed climatology percentage difference, $\Delta\overline{U}_{10}$. (d,e,f) The 90$^{\text{th}}$ percentile wind speed climatology percentage difference, $\Delta U_{10}^{p90}$. (a,d) $\Delta$ = (r4i1p1f1 - r1i1p1f1)/r1i1p1f1, (b,e) $\Delta$ = (r5i1p1f1 - r1i1p1f1)/r1i1p1f1, (c,f) $\Delta$ = (r5i1p1f1 - r4i1p1f1)/r4i1p1f1. As in Figure S1 the hatching in (a, b, c) indicates statistically significant differences, where the p-value is less than 0.05, as determined by a Student t-test. . . . . . . . . . . . . . . . . . . . . . . . . . . . . . . . . . . 4

S3 The 1992–2014 (a) $H_s$ and (b) $H_s^{p90}$, mean and 90$^{\text{th}}$ percentile significant wave height monthly means statistics (23 years x 12 values for each grid cell) in relation to the 2°x2° satellite altimeter (SAT) (represented by a black star) for each WW3/GCM model ensemble member. The ERA5H performance in relation to the SAT dataset over the same period is marked with a black square. The x and y axis represent the standard deviation normalized by the reference SAT standard deviation ($\sigma_{SAT}$). The SAT statistic is placed at the x-axis unit as a reference point. The radial position on the graph represents the Pearson correlation coefficient calculated for each instance of WW3/GCM and ERA5H models against the SAT dataset. The concentric grey circles centered in the black star (x-axis unit) illustrate the Root Mean Square Error (RMSE) values in comparison to SAT. . . . . 4

S4 The 1985–2014 wave climate ensemble performance by ocean climatic regions in relation to ERA5H. (a) IPCC AR6 reference ocean climatic regions[1]: Caribbean (CAR), Southern Ocean (SOO), South Indian Ocean (SIO), Equatorial Indian Ocean (EIO), Bay of Bengal (BOB), Arabian Sea (ARS), South Atlantic Ocean (SAO), Equatorial Atlantic Ocean (EAO), North Atlantic Ocean (NAO), South Pacific Ocean (SPO), Equatorial Pacific Ocean (EPO), North Pacific Ocean (NPO), Arctic Ocean (ARO), South East Asia (SEA), and Mediterranean (MED). (b) The M-score[2] metrics of each WW3/GCM ensemble member computed by averaging the M-score of three integral parameter outputs: $H_s$, $T_{m,02}$, and $\theta$. Each box-plot shows the median ensemble M-score, the Inter-Quartile Range and the minimum and maximum M-score values for each ensemble member. . . . . . . . . 5

S5 The (a, b, c) inter-annual and (d, e, f) inter-ensemble variability of the CMIP6 8-model ensemble mean Significant Wave Height, $\overline{H}_s$, for the periods 1985–2014 and 2071–2100 under SSP1-2.6 and SSP5-8.5 scenarios. Inter-annual variability is computed for each ensemble member as the standard deviation of the yearly means. The resulting standard deviations are then averaged over the entire ensemble to derive the ensemble average inter-annual variability, denoted as $\sigma_Y$. The inter-ensemble variability is determined by computing the standard deviation of each ensemble member 30-year (1985–2014 and 2071–2100) $\overline{H}_s$ climatology (Ens. $\sigma$). . . . . . . . 6

S6 Same as Figure S5 but for the second order spectral mean wave period, $\overline{T}_{m,02}$, climatology. . . . . . . . . . . . . 7

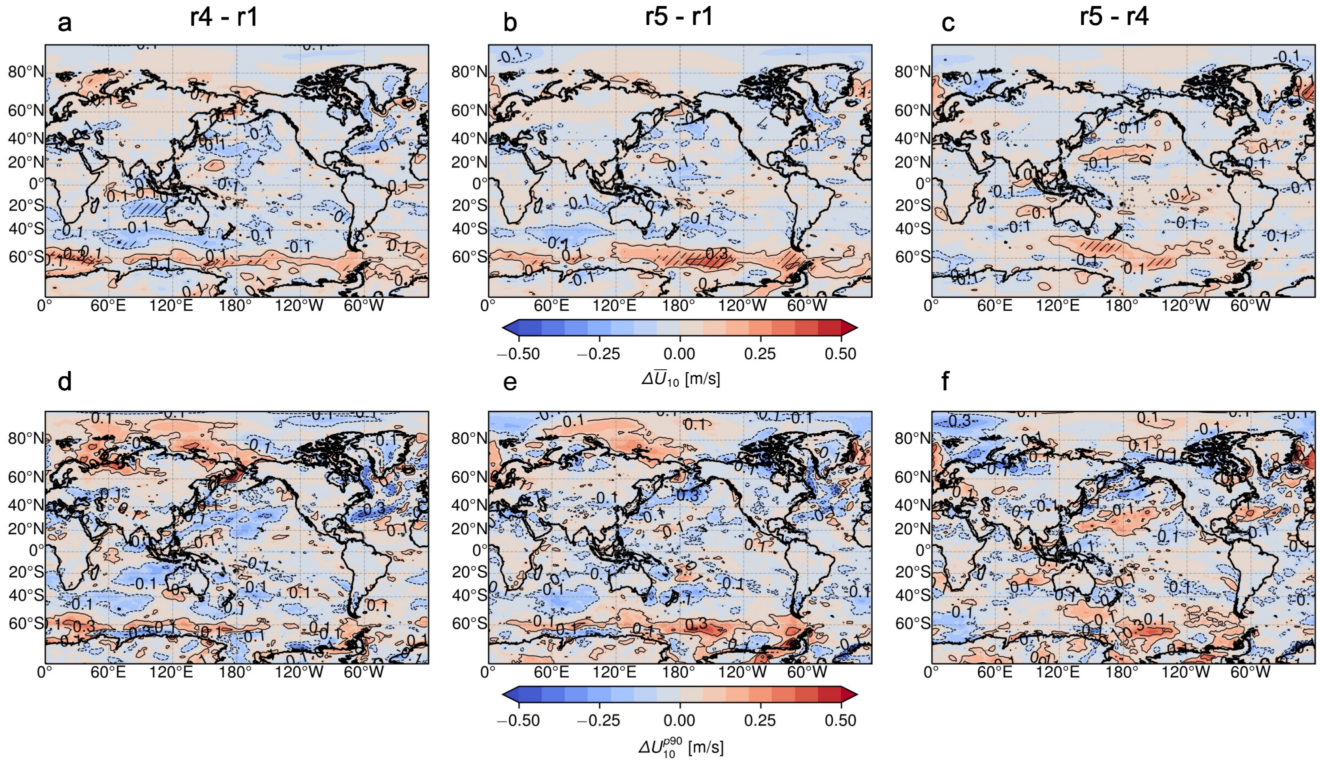


**Figure S1.** Evaluation of the 10-meter surface wind speed, $U_{10}$, climatology from different realizations of the ACCESS-CM2 GCM 1985–2014 CMIP historical experiment. (a,b,c) The average wind speed climatology difference, $\Delta\overline{U}_{10}$. (d,e,f) The 90$^{\text{th}}$ percentile wind speed climatology difference, $\Delta U_{10}^{p90}$. (a,d) Δ = r4i1p1f1 - r1i1p1f1, (b,e) Δ = r5i1p1f1 - r1i1p1f1, (c,f) Δ = r5i1p1f1 - r4i1p1f1. The hatching in (a, b, c) indicates statistically significant differences, where the p-value is less than 0.05, as determined by a Student t-test.

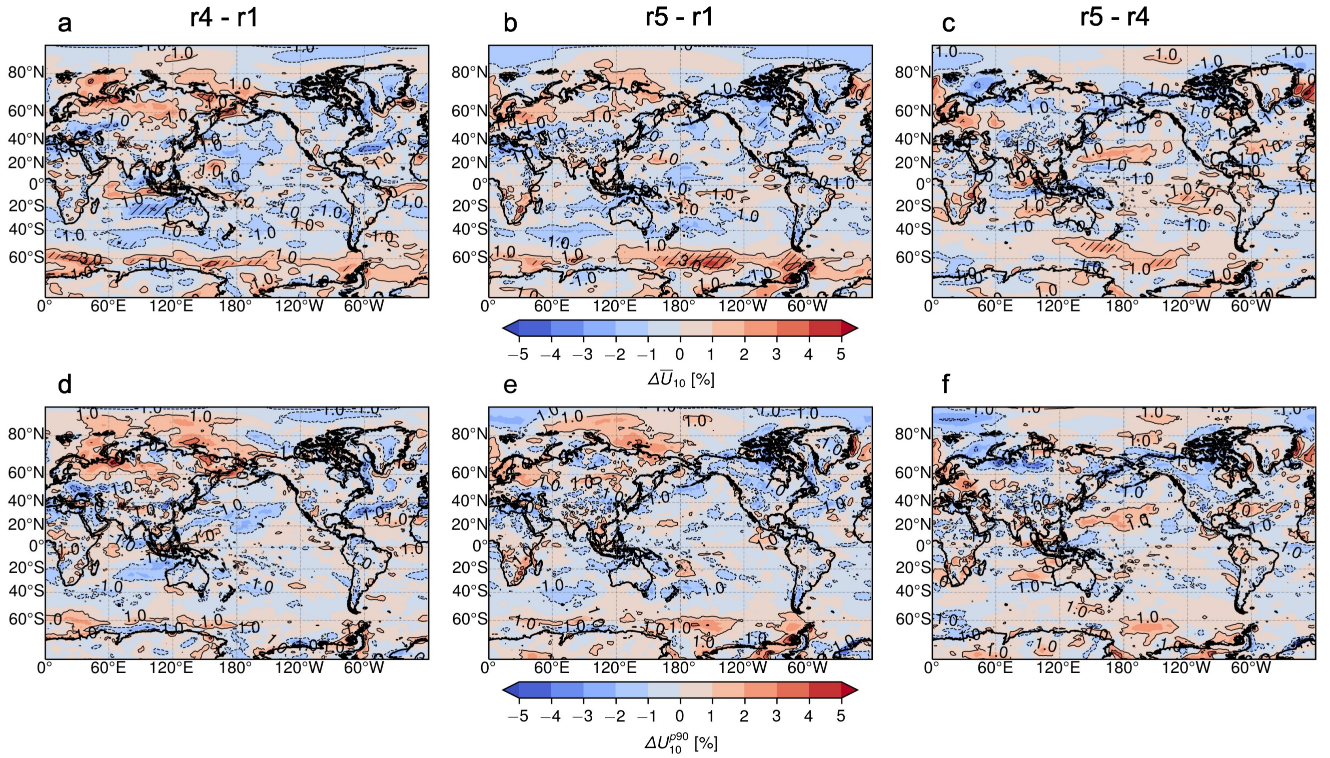


**Figure S2.** Same as Figure S1 but for percentage changes. (a,b,c) The average wind speed climatology percentage difference, $\Delta\overline{U}_{10}$. (d,e,f) The 90th percentile wind speed climatology percentage difference, $\Delta U_{10}^{p90}$. (a,d) Δ = (r4i1p1f1 - r1i1p1f1)/r1i1p1f1, (b,e) Δ = (r5i1p1f1 - r1i1p1f1)/r1i1p1f1, (c,f) Δ = (r5i1p1f1 - r4i1p1f1)/r4i1p1f1. As in Figure S1 the hatching in (a, b, c) indicates statistically significant differences, where the p-value is less than 0.05, as determined by a Student t-test.

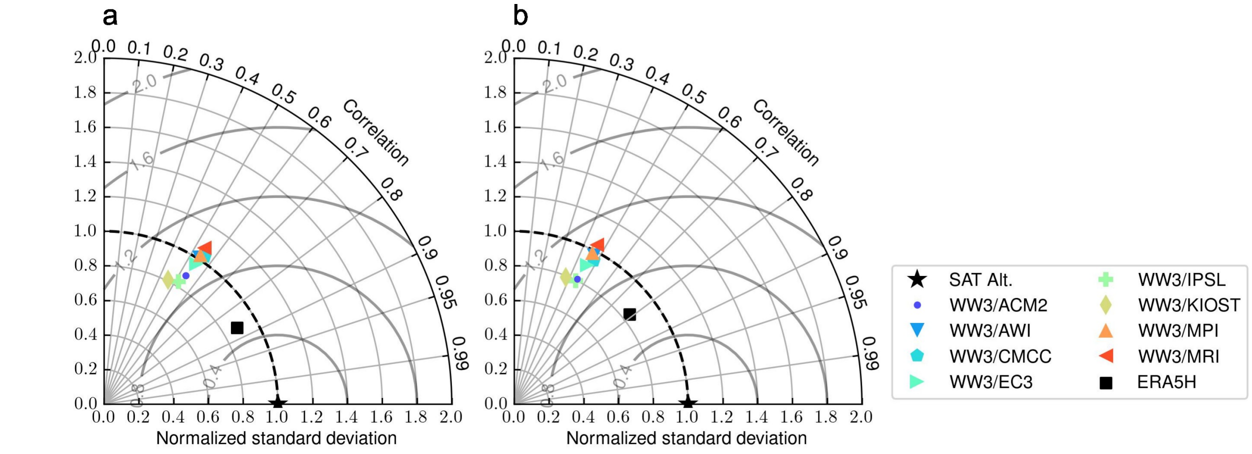


**Figure S3.** The 1992–2014 (a) $H_s$ and (b) $H_s^{p90}$, mean and 90th percentile significant wave height monthly means statistics (23 years x 12 values for each grid cell) in relation to the 2°x2° satellite altimeter (SAT) (represented by a black star) for each WW3/GCM model ensemble member. The ERA5H performance in relation to the SAT dataset over the same period is marked with a black square. The x and y axis represent the standard deviation normalized by the reference SAT standard deviation ($\sigma_{SAT}$). The SAT statistic is placed at the x-axis unit as a reference point. The radial position on the graph represents the Pearson correlation coefficient calculated for each instance of WW3/GCM and ERA5H models against the SAT dataset. The concentric grey circles centered in the black star (x-axis unit) illustrate the Root Mean Square Error (RMSE) values in comparison to SAT.

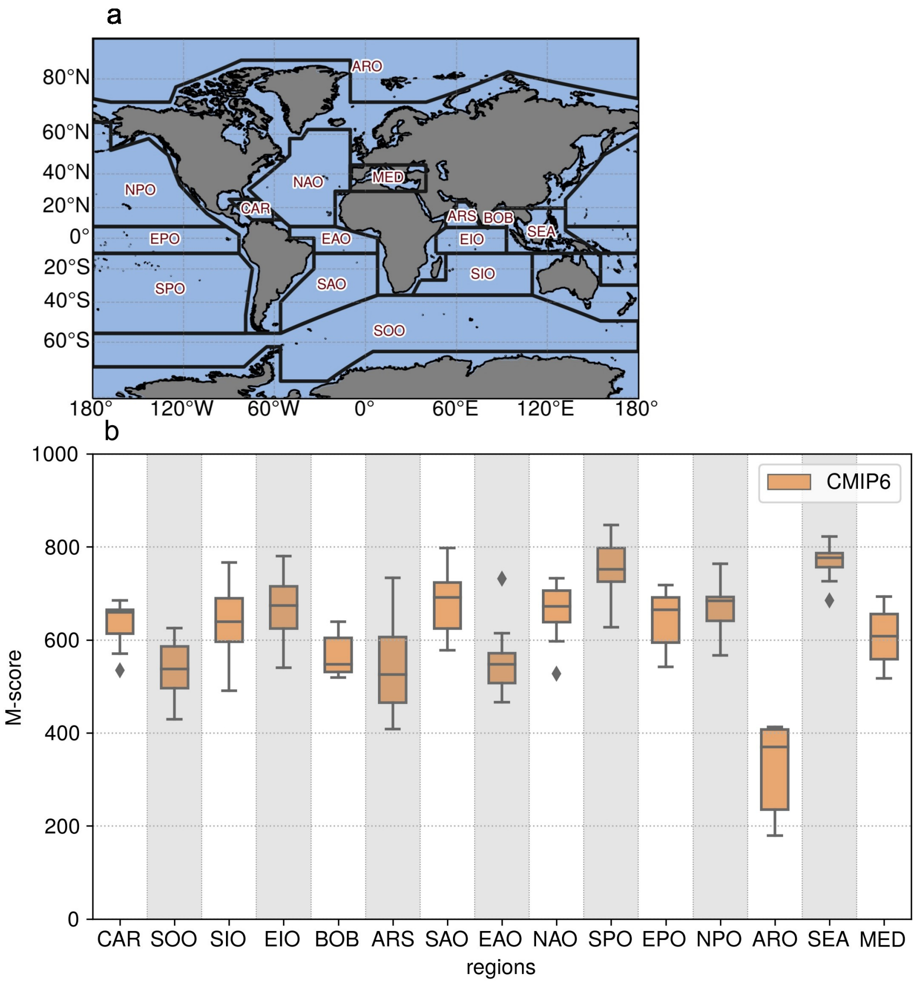


**Figure S4.** The 1985–2014 wave climate ensemble performance by ocean climatic regions in relation to ERA5H. (a) IPCC AR6 reference ocean climatic regions[1]: Caribbean (CAR), Southern Ocean (SOO), South Indian Ocean (SIO), Equatorial Indian Ocean (EIO), Bay of Bengal (BOB), Arabian Sea (ARS), South Atlantic Ocean (SAO), Equatorial Atlantic Ocean (EAO), North Atlantic Ocean (NAO), South Pacific Ocean (SPO), Equatorial Pacific Ocean (EPO), North Pacific Ocean (NPO), Arctic Ocean (ARO), South East Asia (SEA), and Mediterranean (MED). (b) The M-score[2] metrics of each WW3/GCM ensemble member computed by averaging the M-score of three integral parameter outputs: $H_s$, $T_{m,02}$, and $\theta$. Each box-plot shows the median ensemble M-score, the Inter-Quartile Range and the minimum and maximum M-score values for each ensemble member.

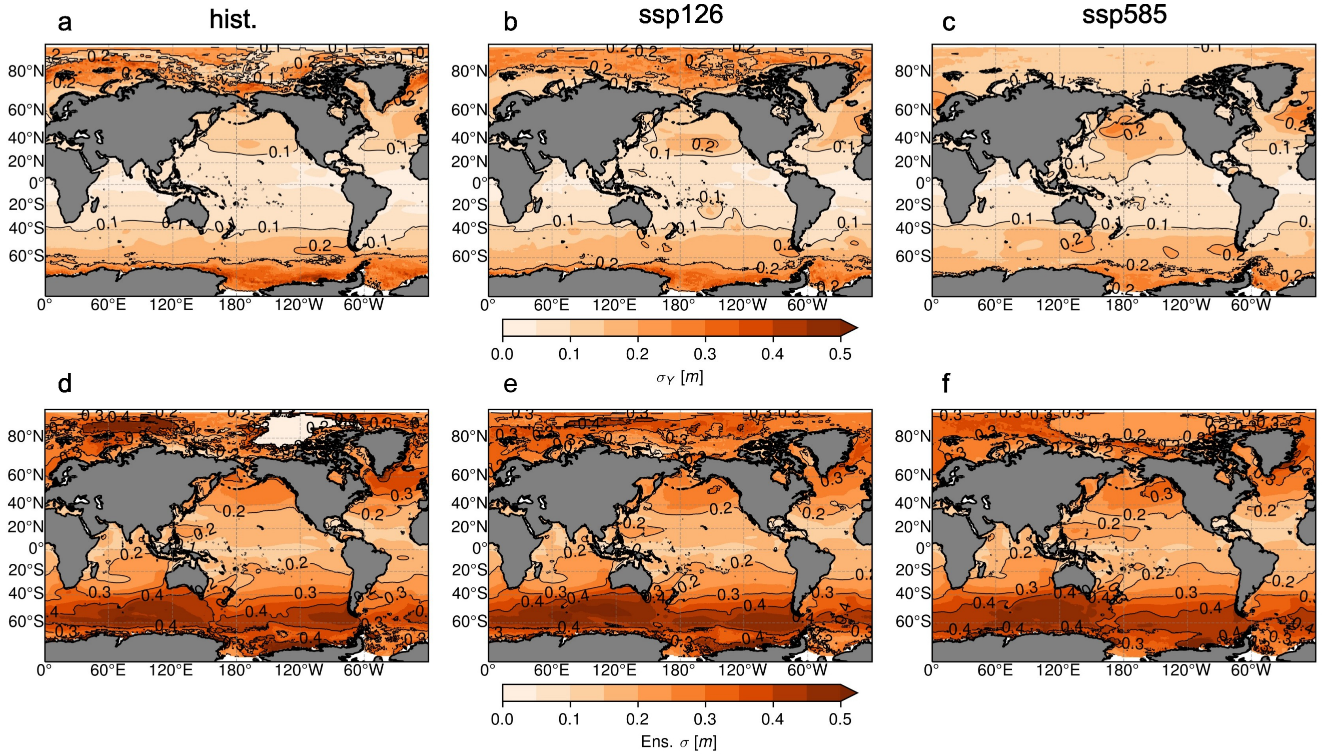


**Figure S5.** The (a, b, c) inter-annual and (d, e, f) inter-ensemble variability of the CMIP6 8-model ensemble mean Significant Wave Height, $\overline{H}_s$, for the periods 1985–2014 and 2071–2100 under SSP1-2.6 and SSP5-8.5 scenarios. Inter-annual variability is computed for each ensemble member as the standard deviation of the yearly means. The resulting standard deviations are then averaged over the entire ensemble to derive the ensemble average inter-annual variability, denoted as $\sigma_Y$. The inter-ensemble variability is determined by computing the standard deviation of each ensemble member 30-year (1985–2014 and 2071–2100) $\overline{H}_s$ climatology (Ens. $\sigma$).

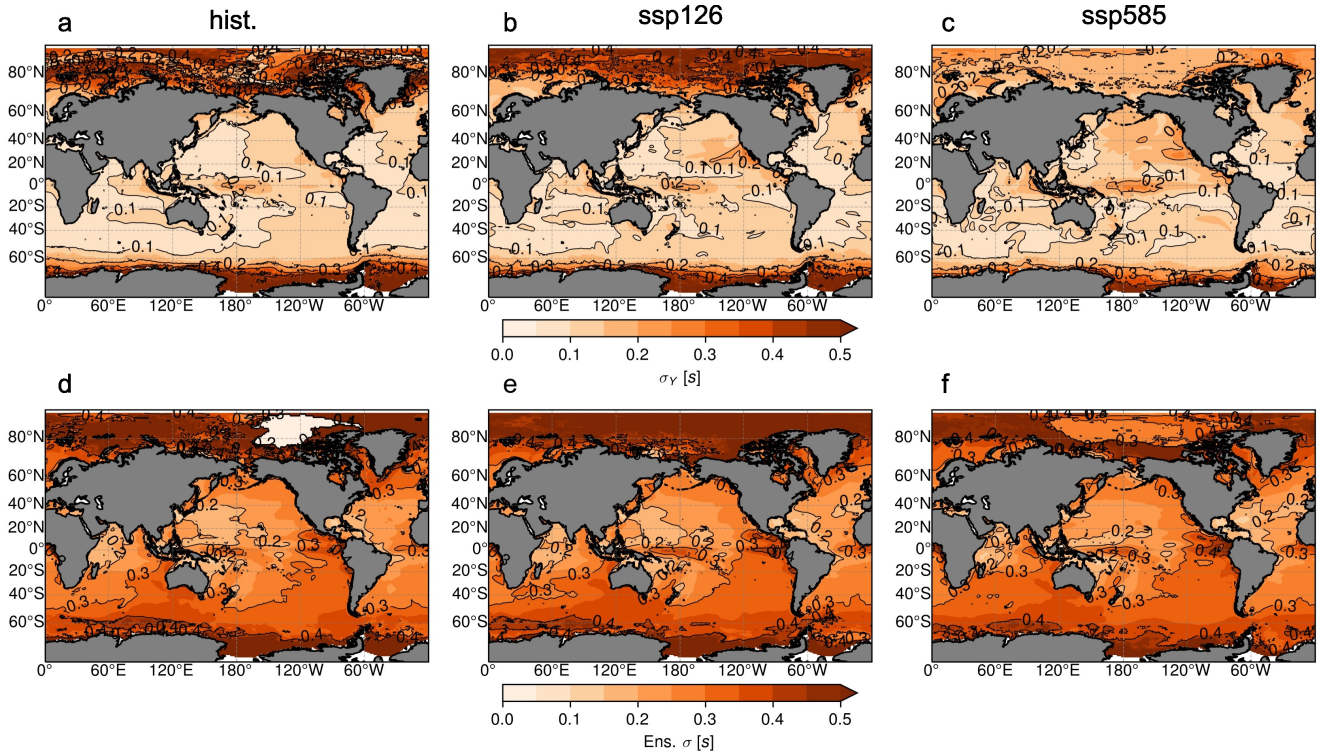


**Figure S6.** Same as Figure S5 but for the second order spectral mean wave period, $\overline{T}_{\mathrm{m},02}$, climatology.

# References

1. Iturbide, M. *et al.* An update of IPCC climate reference regions for subcontinental analysis of climate model data: definition and aggregated datasets. *Earth Syst. Sci. Data* **12**, 2959–2970, https://doi.org/10.5194/essd-12-2959-2020 (2020).

2. Watterson, I. Improved simulation of regional climate by global models with higher resolution: skill scores correlated with grid length. *J. Clim.* **28**, 5985–6000, https://doi.org/10.1175/JCLI-D-14-00702.1 (2015).
